# Dissociable components of visual perceptual learning characterized by non-invasive brain stimulation: Stage 1 Registered Report

**DOI:** 10.1093/braincomms/fcae468

**Published:** 2025-01-02

**Authors:** Marcello Maniglia

**Affiliations:** Department of Psychology, University of California, Riverside, CA 92507, USA

**Keywords:** perceptual learning, brain stimulation, neural plasticity, visual system

## Abstract

Visual perceptual learning (VPL), the training-induced improvement in visual tasks, has long been considered the product of neural plasticity at early and local stages of signal processing. However, recent evidence suggests that multiple networks and mechanisms, including stimulus- and task-specific plasticity, concur in generating VPL. Accordingly, early models of VPL, which characterized learning as being local and mostly involving early sensory areas, such as V1, have been updated to embrace these newfound complexities, acknowledging the involvement on parietal (i.e. intra-parietal sulcus) and frontal (i.e. dorsolateral prefrontal cortex) areas, in aspects concerning decision-making, feedback integration and task structure. However, evidence of multiple brain regions differentially involved in different aspects of learning is thus far mostly correlational, emerging from electrophysiological and neuroimaging techniques. To directly address these multiple components of VPL, we propose to use a causal neuromodulation technique, namely transcranial random noise stimulation, to selectively modulate the activity of different brain regions suggested to be involved in various aspects of learning. Specifically, we will target a region in the occipital cortex, which has been associated with stimulus-specific plasticity, and one in the parietal cortex, which has been associated with task-specific plasticity, in a between-subject design. Measures of transfer of learning to untrained stimuli and tasks will be used to evaluate the role of different regions and test for double dissociations between learning effects and stimulated area, shedding lights on learning mechanisms in the visual system. Evidence of dissociable mechanisms of learning can help refine current models of VPL and may help develop more effective visual training and rehabilitation protocols.

## Teaser

Visual perceptual learning, the training-induced improvement in visual tasks, is attributed to neural plasticity in early processing stages. New evidence suggests involvement of stimulus- and task-specific plasticity. Transcranial random noise stimulation will be used to selectively modulate the activity of brain regions and test their role in in visual perceptual learning.

## Graphical Abstract

**Graphical Abstract:**
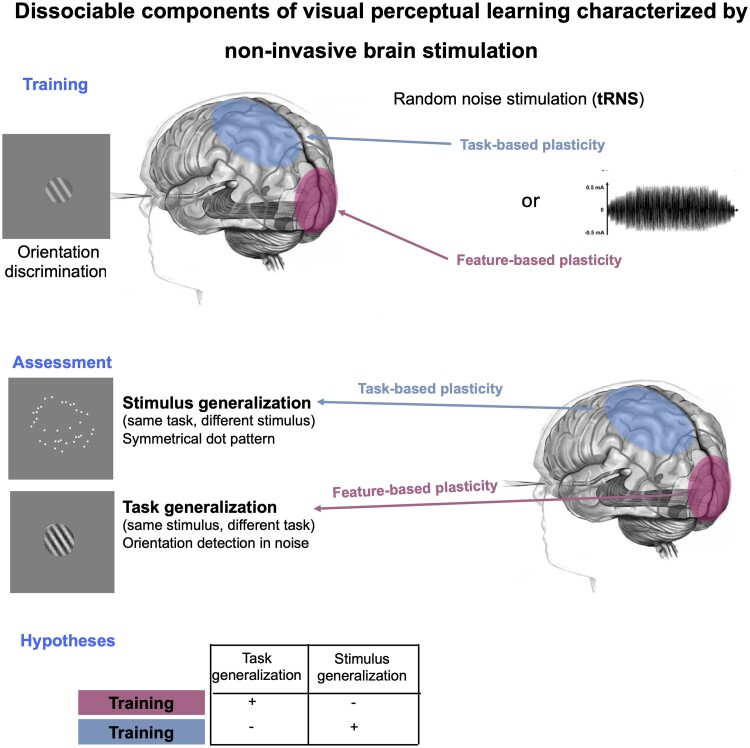

## Introduction

One of the most remarkable features of the human brain is its ability to sharpen perceptual abilities through practice. In the field of vision science, visual perceptual learning (VPL) studies are dedicated to understanding the mechanisms behind training-induced improvement in visual tasks. Early studies on VPL have long considered this phenomenon the result of neural plasticity in early sensory regions, such as the striate visual cortex.^1^ Specifically, early evidence of high learning specificity for characteristics such as retinal location, orientation, spatial frequency or tested eye led to the suggestion that VPL relies on long-term modification at early stages of visual analysis, whose neural substrates are found in the early visual cortex.^2-5^

This initial hypothesis received apparent support from early VPL electrophysiology studies, which showed changes in functional properties of neurons in early visual area V1 following orientation discrimination training,^6,7^ thus further pointing towards neural substrates of VPL corresponding to local and specific stimulus processing loci.

However, more recent evidence of learning generalization beyond the initial constrains of spatial frequency, orientation or retinal location (e.g.^8-10^) challenges the hypothesis that the early visual cortex is the (sole) neural substrate of VPL: a number of studies showed that training on classic VPL paradigms can lead to orientation, location and even task transfer, if basic training paradigms are altered to, for example, include ‘dummy trials’ that do not contain the target,^11^ use ‘double training’ paradigms,^9,12^ or by manipulations of exogenous attention,^13^ or by systematically varying multiple stimulus parameters such as orientation, spatial frequency and location.^14^

These findings prompted the field of VPL to reconsider the initial finding of learning specificity, viewing it as a potential by-product of the methods and paradigms employed in early studies, characterized by a litany of trials with unchanged stimulus characteristics, rather than an inherent, and unescapable, characteristic of VPL. In the context of this conceptual re-framing, it has been proposed that learning specificity observed in early VPL studies might have emerged because of the absence of bottom-up stimulation and top-down modulation at the untrained conditions,^15^ which suggests the involvement of multiple mechanisms and, possibly, neural substrates in generating learning and transfer.^16^

This idea is supported by recent neuroimaging and electrophysiology evidence that suggests that VPL can be associated with different types of neural plasticity, manifesting in distinct anatomical regions such as parietal^17^ and frontal areas.^18^ These regions appear to undergo training-related changes, which appear distinctively associated with task representation,^17,18^ in contrast to occipital changes that are associated to stimulus representation.^6,7,17^ Specifically, Shibata and colleagues^17^ showed that VPL of motion direction discrimination is associated with at least two types of neural plasticity, pertaining to characteristics of the stimulus (feature-based plasticity) and observed in a sensory area (V3) and characteristics of the training task (task-based plasticity) observed in an associative area (the intra-parietal sulcus within the posterior parietal cortex).

This mounting evidence led the field of VPL to embrace a tale of increasing complexity, with recent models and framework of VPL^16,19-21^ describing learning as the product of different regions and mechanisms depending on the characteristics of the stimulus and the needs of the task. This, alongside more recent studies and models incorporating multiple mechanisms and brain regions involved in learning,^16,18,19^ suggest that learning outcomes in visual training studies can potentially arise from multiple neural plasticity mechanisms. Crucially, this new way of looking at plasticity and generalization opens exciting translational possibilities to unlock the full potential of VPL, which could lead to real-life applications, for example in clinical practice.^22,23^ Consequently, VPL stands poised to emerge as powerful tool for understanding and promoting neural plasticity in the whole brain.^16^

However, most of this evidence is correlational in nature, and the question remains whether the observed neuroimaging and electrophysiology results are indeed manifestations of such learning mechanisms.

To answer this question, here we plan to utilize non-invasive electric stimulation to selectively modulate the activity of targeted regions while participants are engaged in a VPL task. Specifically, we plan to selectively boost different VPL components by using transcranial random noise stimulation (tRNS), a form of non-invasive brain stimulation that has shown effectiveness in modulating cortical plasticity to produce behavioural benefits in visual perception, both in isolation to induce transient improvements,^24^ and paired with visual training to obtain more long-lasting effects.^25-32^

tRNS is a type of transcranial electrical stimulation (tES) protocol, in which a weak current (usually 0.5–2 mA) oscillating at random frequencies (0.1–640 Hz) is delivered through the scalp using a pair of electrodes.^33^ Differently from more standard tES protocols, such as transcranial direct current stimulation, tRNS does not produce differential effects between the two electrodes, since the current continuously changes polarity between the two electrodes.^33^ However, unlike other forms of alternate current protocols, such as transcranial alternate current stimulation, the change in polarity is not applied at a specific frequency but rather randomly within a range,^34^ thus preventing neuronal entrainment. Importantly, frequency range and amplitude parameters need to be chosen carefully, as previous studies have showed that higher frequency ranges^35^ and wide range of overall frequencies^36^ appear necessary to optimize tRNS effects.

tRNS holds significant potential as a tool to understand mechanisms of learning and causally test hypotheses on the functioning of the learning brain that emerged from neuroimaging and electrophysiology, correlational techniques. Recent evidence showed that online tRNS improves contrast sensitivity for oriented grating,^37^ and, when paired with crowding reduction training,^28^ leads to larger learning effects with respect to sham (placebo) stimulation. In studies comparing tRNS with other stimulation protocols, tRNS seems to lead to larger cortical excitability^38^ and training effects.^29^

However, currently, its use in the context of VPL appears mostly confined to increase cortical excitability in the occipital cortex to boost learning effects.^25,27,28,32,39^ Moreover, most of these studies look at transfer of learning in terms of performance improvement, thus their chosen transfer tasks may not be optimal for characterizing the mechanisms underlying learning. Transfer of learning could provide valuable insight into the level at which learning takes place.^17^ Indeed, through transfer assessments, we can directly test hypotheses concerning the role that specific cortical regions play in different aspects of the learning process.

Here, we plan to put to test the hypothesis that tRNS can selectively boost task- and stimulus-related plasticity in standard VPL task, orientation discrimination, as assessed by transfer tasks looking specifically at stimulus and task components. We hypothesize that boosting task- and feature-related plasticity with tRNS will promote transfer of learning to untrained stimuli and tasks, respectively.

Specifically, boosting stimulus-related components through early visual cortex (occipital) stimulation is expected to generalize learning to an untrained task that uses the same stimulus (orientation detection with Gabor patches). On the other hand, boosting task-related component by stimulating higher-level regions (posterior parietal cortex) involved in attentional,^40^ decision-making and task processing^17^ is expected to generalize learning to untrained stimuli used in the same task (orientation discrimination with symmetrical dot patterns, which have been found to be processed in extra-striate regions,^41,42^ unlike the Gabor patches in the training protocol, whose processing is mainly within the striate visual area). Causal evidence of differential involvement of brain regions in different aspects of VPL can help inform and develop more sophisticated models of VPL, broaden our understanding of mechanisms of VPL, and potentially lead to increased translational opportunities for these protocols.

The overarching idea of this proposal is thus to use brain stimulation during training to selectively boost different learning components, namely stimulus-related plasticity through occipital stimulation, and task-related plasticity through parietal stimulation, while participants are engaged in a standard VPL task (orientation discrimination).

Hypothesis 1 is that occipital stimulation would significantly increase stimulus-related plasticity, as assessed by transfer of learning task assessments in which participants are tested with the same training stimulus (Gabor patches) in a different task (orientation detection^17^).

Hypothesis 2 is that parietal stimulation would significantly increase task-related plasticity (and possibly attentional gain^40^ and decision-making processes^43^), as assessed by transfer of learning task assessments in which participants are tested with the same training task (orientation discrimination) using a different stimulus (symmetrical dot patterns^15^) known to be processed by different neural substrates with respect to the training stimulus.

The two hypotheses tested here are complementary, and the goal of the statistical analyses is to test a double dissociation, in which the group undergoing occipital stimulation and the group undergoing parietal stimulation would show significant transfer in one of the assessment conditions but not in the other.

Additionally, the experimental setup can allow use to test a further, exploratory hypothesis concerning transfer of learning. Specifically, there is evidence suggesting that parietal tRNS combined with VPL leads to improvements in visual attention tasks,^40^ and explicitly engaging attention during VPL has been shown to promote retinal generalization.^13^ Similarly, occipital stimulation seems to promote retinal generalization when combined with VPL.^28^ Thus, in the context of the general design proposed here, we believe it worth testing this additional hypothesis concerning the role of occipital and/or parietal stimulation in promoting retinal transfer, and whether the magnitude of this effect is similar or different between stimulation conditions.

## Materials and methods

**Figure 1:**
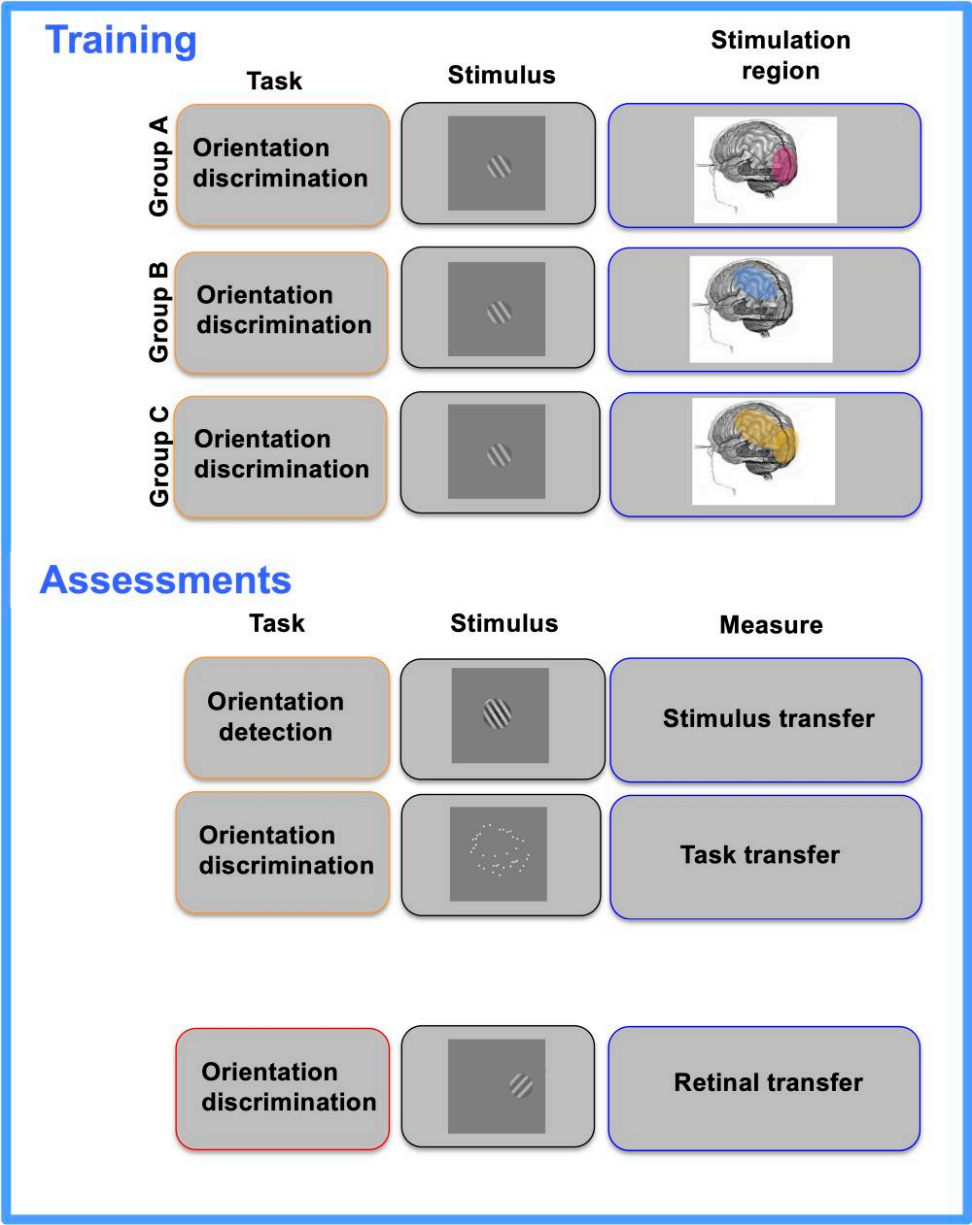
**Experimental layout.** Participants will perform four assessment tasks measuring stimulus transfer (orientation detection on the trained stimulus, a Gabor patch, embedded in noise) task transfer (performing the training task on an untrained stimulus, symmetrical dot patterns) and retinal transfer (training task on the training stimulus in an untrained retinal location). During training, Gabor patches will be presented 4° in the periphery. Stimuli will be presented in the same hemifield. The reference orientation Gabor (36° or 126°, counterbalanced) and the test orientation Gabor (reference + Δori) will be presented in two stimulus intervals (100 ms) in a random order, with 500 ms ISI. The observers will judge in which stimulus interval the Gabor was more clockwise. Group A will be training with concomitant occipital (‘Early visual cortex’ in the figure) stimulation (boosting stimulus processing^17^), Group B will be trained with concomitant posterior parietal cortex (PPC) stimulation (boosting task-related processing/attention^17^), and Group C will be trained with concomitant sham (placebo) stimulation, as control group. A video eye tracker will be used to control for eye movements.

**Figure 2:**
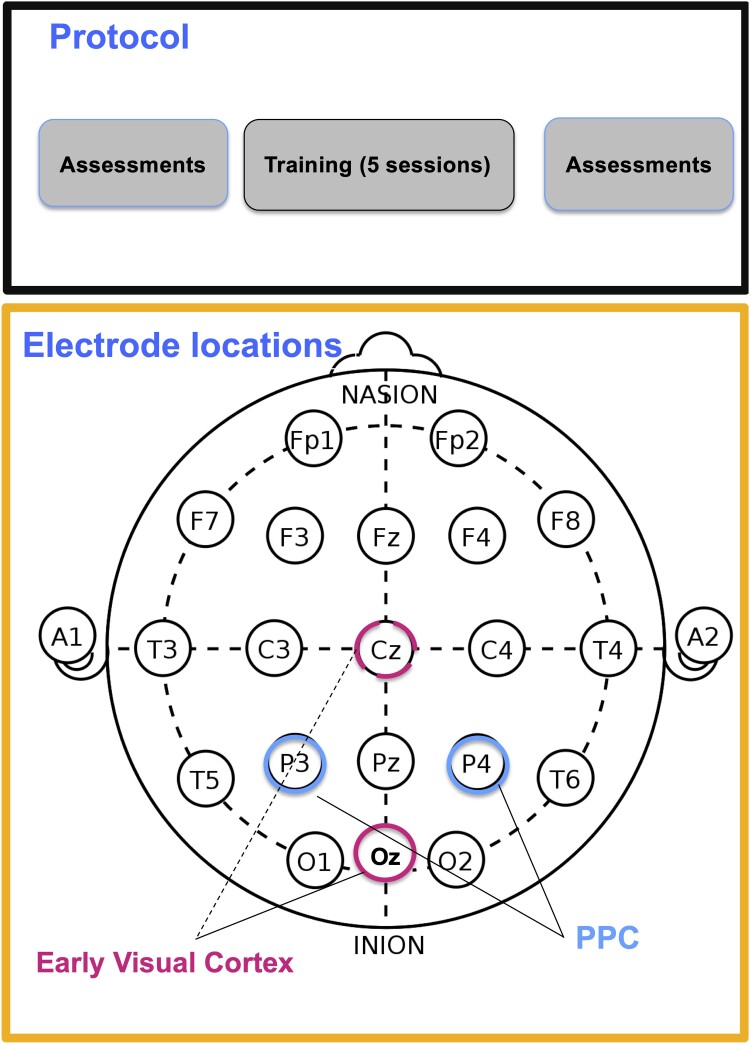
**Protocol and electrode locations.** Participants will be trained for five sessions. Before and after training, assessment tasks will be conducted to test transfer effects. Three brain regions will be targeted, in a between-group design: ‘Early visual cortex’, with active electrode around OZ and reference electrode on Cz;^28,37,44^ posterior parietal cortex (PPC) with bilateral stimulation on P3 and P4,^40^ and sham (placebo) stimulation.

The experimental design is summarized in Figs 1 and 2.

### Participants

Participants for the study will be recruited among the student body of UCR campus. Exclusion criteria are vision worse than 20/40. Participants will be randomly assigned to one of the three experimental conditions described below (randomization using a random sequence generated through MATLAB). The proposed research complies with all relevant ethical regulations of the University of California Riverside; The IRB approval is currently pending. Written informed consent will be obtained from all human participants. Participants will be compensated $10 per hour for their participation in the study.

Funds are available to start recruitment and data collection upon acceptance of the registered report. The study will be registered on ClinicalTrials.gov upon acceptance of the registered report. Testing will be conducted in the UCR Department of Psychology building.

### Training

All participants in the three groups will perform the same training type. The task will be Orientation discrimination. Gabor patches will be presented on a Display++ (Cambridge Research Systems) 4° in the periphery. Stimuli will always be presented in the same hemifield, with online eyetracking (TRACKPixx; VPixx Technologies) to control eye movement (stimulus location gaze-contingent). The spatial frequency of the Gabor patch will be of 6 cpd, as a compromise between evidence of tRNS efficacy in enhancing detection of low contrast Gabor patches of high spatial frequency^37^ and the peripheral presentation of the stimuli. Similar to,^15^ the sigma of the Gaussian envelop will be of 0.67°. The fixation cross will be first presented for 300 ms and a blank gap of 250 ms will follow, before the onset of the first stimulus interval. The reference orientation Gabor (36° or 126°, counterbalanced among observers) and the test orientation Gabor (reference + Δori) will be presented in two stimulus intervals (100 ms each) in a random order, separated by a 500 ms inter-stimulus interval. The observers will judge in which stimulus interval the Gabor was more clockwise. Auditory feedback will be given on incorrect responses.^45^

#### Group A

This group will be trained on orientation discrimination with concomitant occipital (OZ) stimulation, aiming at boosting stimulus processing as in previous studies.^17,28,37^

#### Group B

This group will be trained on orientation discrimination with concomitant parietal (P3/P4^46^) stimulation, aiming at boosting task-related processing/attention.^40^ Previous studies showed involvement of parietal regions, in particular the intra-parietal sulcus in VPL of global motion,^47^ and the corresponding area in non-human primate, LIP,^48,49^ similarly involved in perceptual decision-making task on global motion^50,51^ and VPL of global motion.^51,52^ Additionally, evidence shows that the posterior parietal cortex is related to dorsal and ventral attention network^40^ and spatial attention,^53^ which can play a role in task-related plasticity.

#### Group C: training on orientation discrimination with sham stimulation (control group)

Specifically, participants will undergo the same montage as Groups A and B (counterbalanced and randomized between occipital and parietal montage across participants), as sham stimulation is delivered (see details below).

### Transfer

All participants in the three groups will perform the same transfer tasks.

#### Transfer of stimulus: orientation detection (Gabor in noise)

Following Shibata *et al*.^54^ and Tan *et al*.,^55^ Gabor patches will be spatially masked by a noise pattern using a pixel substitution method.^56,57^ Noise fields will be generated from a sinusoidal luminance distribution at a given signal-to-noise (S/N) ratio. For example, in the case of a 10% S/N ratio, 90% of the pixels of the Gabor patch will be replaced with the noise pattern. The orientation of the Gabor patch will be the same as during training. Participants will perform a two-interval forced-choice orientation-detection task, in which one stimulus interval contains a Gabor patch with a certain S/N ratio and the other stimulus interval contains only noise (0% S/N ratio). Each trial will start with a 500 ms fixation interval. After two 50 ms stimulus intervals separated by a 300 ms inter-stimulus interval, participants will be asked to report which stimulus interval contained the Gabor patch.

#### Transfer of task

Orientation discrimination (from reference) with symmetrical dot patterns.^15^ Following,^15^ the symmetric dot patterns will consist of 18 pairs of bilaterally symmetric white dots (0.1° diameter), confined to an area divided into 18 × 18 invisible square compartments (0.16° × 0.16° each). Constrains described in Wang *et al*.^15^ will be used to generate the patterns. Participants will be presented with a distribution of dots mirrored along an imaginary central axis, providing the (tilted) centre of the configuration. They will be asked to report whether the second configuration’s axis of symmetry is tilted more clockwise or counterclockwise with respect to the reference pattern. In each trial, the configuration will be generated anew. Processing of symmetric pattern is known to engage different visual cortical areas than Gabor patches: grating orientation is encoded in V1,^58,59^ whereas symmetric dot patterns only selectively activate higher-order cortical areas.^41,42^

#### Transfer of retinal location

Participants will undergo one additional transfer task to test an exploratory hypothesis concerning generalization effect for the two types of stimulation regions. Specifically, participants will perform the trained task in their untrained hemifield (symmetrical location in the visual field with respect to the trained side).

### Stimulation protocol

tRNS is a form of alternating current of random with frequencies of fluctuation distributed across a range of 100–640 Hz with zero-mean e.g.^29^ tRNS will be delivered using a battery-driven stimulator (BrainSTIM, EMS) through a pair of saline-soaked sponge electrodes.

The total duration of stimulation will be 30 min to cover the entire training session. The stimulation will be turned on a few minutes before starting the session to optimize cortical excitability effects.^60^ In the occipital stimulation condition, the active electrode will have an area of 20 cm^2^ and will be placed over the occipital cortex measured at 3 cm above the inion.^28,29,44,61^ The reference electrode will have an area of 49 cm^2^ and will be placed on the vertex. The larger size of the reference electrode is intended to make it inert due to low current density.^62,63^ In the parietal stimulation condition, both electrodes will have an area of 20 cm^2^. Importantly, tES is considered to be safe, tolerable, and acceptable to use in adults.^64-68^ Additionally, the current density will be maintained well below the safety limits (always below 1 A/m^2^;^65^). We will follow tES guidelines (i.e.^67^) and conduct questionnaires on adverse effects of tES to contribute to the growing body of research on this subject. We believe that a larger database over multiple protocols will help improve safety and reduce adverse effects of tES. The electrodes will be kept in place with non-conductive elastic bandages. To better understand the diffusion of the current through the cortex and the size of the stimulated area according to this setup, we will calculate and visualize the expected current density with the SimNibs software,^69^ to confirm that the current density is mostly localized in early visual areas. We will use a fixed intensity value of 1 mA, as a compromise between van der Groen and Wenderoth^70^ that showed that an intensity between 0.5 and 1 mA produces the largest increase in sensory enhancement, compared to a non-stimulation condition, when participants were presented with subthreshold stimuli (threshold at 60%, consistent with our targeted thresholds during training), and evidence of lack of cortical excitability effects for stimulation intensities lower than 1 mA.^71^ Estimation of average EF for stimulation type, location and intensity will be generated using toolboxes such as SimNIBS.

#### Occipital transcranial random noise stimulation

Following previous studies using tRNS to target early visual cortex,^28,32,37,61^ the active electrode will be placed over the occipital cortex, measured at 10% of the individual inion–nasion distance (∼3 cm) above the inion (around Oz). The reference electrode will be placed on the vertex (Cz).

#### Parietal transcranial random noise stimulation

Following previous studies using tRNS to target PPC^40^ electrodes will be placed on P3 and P4.

#### Control montage

Similar to previous studies, control condition will be a sham (placebo) montage, following the occipital and parietal montage, but with the current ramping up and down within the initial 30 s of stimulation and then again within the last 30 s of stimulation. sham (placebo) stimulation can be applied in a way that is indistinguishable from active stimulation.^72^ Sham stimulation is carried out by increasing current over several seconds to the target strength, and then tapering off over several seconds. Using this approach, participants may experience itching and tingling that would be virtually indistinguishable from the active stimulation. During active tES stimulation, sensations are transient because the participants accommodate to the current, whereas during sham stimulation, sensations fade due to the current being tapered off.

### Power analysis

Power analyses conducted on conducted on the effect sizes reported in studies using VPL and tES^27-30,39^ would predict a sample size up to 15 participants per group. These were calculated using G*Power and based on expected differences between training types (i.e. expectation that tRNS will produce larger training effects in orientation discrimination than controls, based on the effect size *η*^2^ = 0.14 reported in Contemori *et al*.^28^ and expected transfer effects to low level visual functions (i.e. expectation that visual acuity thresholds would significantly improve after contrast detection training, based on the effect sizes of *η*^2^ = 0.25 reported in Moret *et al*.^31^). However, we believe that several studies in the field of VPL tend to be underpowered, and this is even more true for studies comparing multiple montages and outcomes measures, given the novelty of this approach. Thus, given our additional interest in increasing rigour in the study of training effects, we decided to err on the side of caution and plan to have 20 participants per group, for a total of 60.

Data exclusion criteria will follow standard outlier rejection procedures (i.e. 3 SD above or below the mean).

### Statistical analysis plan

Our main analysis will focus on transfer performance, while two exploratory analyses will look at both training and transfer performance. Data collection and analysis will be performed blind to the conditions of the experiments. A summary of the research questions of this proposal is shown in Supplementary Table 1.

#### Effects of stimulated region on training components transfer

Our overarching hypothesis is that training that targets different brain regions gives rise to different patterns of learning, as assessed by performance in transfer tasks addressing task- and stimulus-related learning. Tests of this hypothesis will involve a mixed-model ANOVA on transfer index (learning in the training/learning in the transfer) with between-subject factor stimulation (occipital versus parietal versus control) and within-subject factor transfer (stimulus versus task). In this context, transfer index (TI) is defined as TI = [improvement at the transfer condition/improvement at the trained condition], in which TI = 0 would indicate complete learning specificity and TI = 1 would point towards complete learning transfer.

*Post hoc* analyses will be conducted to verify our hypotheses, specifically, that the occipital group will show significant transfer of learning to the stimulus transfer assessment task, and the parietal group will show significant transfer of learning to the task transfer assessment task. Supplementary Table 1 shows specific hypotheses on transfer outcomes.

#### Effects of stimulated region on retinal transfer

In this exploratory analysis, we test whether stimulating parietal regions, who have been shown to be involved in aspects of attention,^40^ would promote transfer of learning to other retinal locations. The rational is that attentional improvements might be less location-specific than sensory improvements due to occipital stimulation.

#### Effects of stimulated region on training

The second exploratory analysis will be mixed-model ANOVA, with one between-subject condition stimulation (occipital versus parietal versus control) and one within-subject condition t(session 1 versus session 2 versus session 3). This analysis is intended as exploratory and aimed at characterizing training effect across stimulation regions. Previous results suggest that stimulating occipital cortex leads to larger training effects with respect to control regions.

In addition to that, Bayes factors,^73^ the ratio of the likelihood of one particular hypothesis to the likelihood of another, will be used to determine the probability of the null hypothesis compared to the alternative hypothesis. Bayes factors > 3 indicate substantial evidence for the alternative hypothesis and values < 0.33 indicate substantial evidence for the null hypothesis.

## Supplementary Material

fcae468_Supplementary_Data

## Data Availability

Raw data and materials will be shared on public repository, such as Mendeley or GitHub, upon acceptance of the manuscript. Code, in the form of MATLAB scripts, will be shared on public repositories, such as Mendeley or GitHub, upon acceptance of the manuscript.
