# Supplementary material for: Dissociable components of visual perceptual learning characterized by non-invasive brain stimulation: Stage 1 Registered Report
Source: Brain Commun. 2025 Jan 2;7(1):fcae468. doi: 10.1093/braincomms/fcae468 (PMC11694700; doi:10.1093/braincomms/fcae468)
Supplement: fcae468_Supplementary_Data [file fcae468_supplementary_data.docx]

**Dissociable components of visual perceptual learning characterized by non-invasive brain stimulation: Stage 1 Registered Report**

Marcello Maniglia*

Department of Psychology, University of California, Riverside, Riverside, CA, USA

*mmanig@ucr.edu

# Supplementary Material

# Supplementary Table 1. Design Table summarizing the research questions.

| **Question** | **Hypothesis** | **Sampling plan (e.g. power analysis)** | **Analysis Plan** | **Interpretation given to different outcomes** |
| --- | --- | --- | --- | --- |
| Is the occipital cortex causally involved in stimulus-related plasticity during perceptual learning? | Stimulating the occipital cortex during training enhances stimulus-related plasticity | Sixty-six undergraduate Psychology students will be initially recruited with approximately 10% attrition expected, given the short duration of the study | Evaluate transfer index for the stimulus transfer conditions. We expect significantly larger transfer to orientation detection for the occipital stimulation group with respect to both the parietal stimulation and the control group. | Transfer might fail due to multiple reasons, including stimulation intensity, duration of training, etc |
| Is the posterior parietal cortex causally involved in task-related plasticity during perceptual learning? | Stimulating the posterior parietal cortex during training enhances task-related plasticity | This question will be addressed within the same experimental design as Question 1, thus data from the same participants will be used | Evaluate transfer index for the task transfer condition. We expect significantly larger transfer to orientation discrimination with symmetrical dot patterns for the parietal stimulation group with respect to both the occipital stimulation and the control group. | Transfer might fail due to multiple reasons, including stimulation intensity, duration of training, etc |
| Is the occipital cortex causally involved in transfer of learning to untrained location? | Stimulating the occipital cortex during training enhances retinal location transfer | This exploratory question will be addressed within the same experimental design as Question 1 and 2, thus data from the same participants will be used | Evaluate transfer index for the stimulus transfer conditions. We might expect significantly larger transfer to untrained retinal location with respect to the control group. | Occipital cortex stimulation might be insufficient to cause a complete transfer of learning to untrained retinal location, as non-retinal components, such as task- and attentional-related ones, are preferentially processed outside of the early visual cortex |
| Is the posterior parietal cortex causally involved in transfer of learning to untrained location? | Stimulating the posterior parietal cortex during training enhances retinal location transfer | This exploratory question will be addressed within the same experimental design as Question 1 and 2, thus data from the same participants will be used | Evaluate transfer index for the stimulus transfer conditions. We might expect significantly larger transfer to untrained retinal location with respect to the control group. | Parietal cortex stimulation might be insufficient to cause a complete transfer of learning to untrained retinal location, as its involvement in VPL might still rely on retinal-specific processing |
